# Papilla‐Preserving Gingival Fiber Retention Osseous Surgery in the Anterior Maxilla: A Novel Clinical Technique

**DOI:** 10.1111/jerd.70226

**Published:** 2026-07-02

**Authors:** Abdusalam E. Alrmali, Hom‐Lay Wang

**Affiliations:** ^1^ Department of Periodontics and Oral Medicine School of Dentistry, University of Michigan School of Dentistry Ann Arbor Michigan USA; ^2^ Department of Oral Medicine, Oral Pathology, Oral and Maxillofacial Surgery University of Tripoli School of Dentistry Tripoli Libya

**Keywords:** dental, esthetics, periodontal pockets, periodontics, periodontitis, surgical flaps

## Abstract

**Objective:**

To describe papilla‐preserving gingival fiber retention osseous surgery (PP‐FibReOS), a biologically driven resective approach intended to eliminate periodontal pockets while maintaining interproximal connective tissue and papillary stability in esthetically demanding maxillary anterior sites.

**Clinical Technique:**

PP‐FibReOS was implemented in two challenging anterior maxillary scenarios: functional crown lengthening and surgical pocket elimination in a stage IV, grade C periodontitis case. The key feature of this protocol uses a split‐full‐split buccal flap with selective coronal tissue removal, combined with deliberate preservation of a de‐epithelialized interproximal supracrestal fiber complex attached to the palatal flap, which is designed to support papillary volume during healing. Osseous recontouring is performed conservatively, with the flap repositioned in a double‐layered papillary complex closure just coronal to the bone crest. At 36‐month follow‐up, both cases showed shallow probing pocket depths (PPD) of 1–3 mm and absence of bleeding on probing, together with stable interdental papilla positions, high clinician‐reported esthetic scores (pink esthetic score [PES] 11–12), and patient satisfaction ratings of 10/10.

**Conclusion:**

Within the limits of this two‐case proof‐of‐concept report, PP‐FibReOS may offer a promising alternative for maxillary resective surgery when preservation of papillary architecture is a primary objective. Larger controlled studies are needed to clarify its indications, limitations, and long‐term predictability.

**Clinical Significance:**

PP‐FibReOS provides a possible approach for selected anterior maxillary defects in which pocket elimination or functional crown lengthening must be balanced against preservation of interdental soft‐tissue dimensions. In patients with a thick periodontal phenotype, adequate supracrestal connective tissue, and predominantly horizontal or shallow angular defects, a fiber‐retentive palatal flap combined with conservative osseous reshaping may help maintain stable, shallow probing pocket depths while limiting increases in clinical crown length and visible loss of papillary fill.

## 1 Introduction

Periodontal surgical therapy aims to establish a healthy, maintainable periodontium and favorable restorative conditions by eliminating pathologic pockets or creating adequate supragingival tooth structure, while preserving soft‐tissue support and esthetics, particularly in the maxillary anterior region [1, 2, 3]. Classical resective approaches, such as apically positioned flap (APF) surgery with osseous recontouring, are effective in eliminating periodontal pockets and establishing a positive bony architecture, or in providing the clinical crown length required for prosthetic rehabilitation, but often at the cost of increased clinical crown length and loss of interdental papillae [4, 5, 6, 7]. These esthetic sequelae are especially problematic in patients with high smile lines or triangular tooth forms, where even limited recession can result in visible black triangles.

Consequently, techniques that allow pocket elimination or functional crown lengthening while preserving supracrestal connective tissue (SCT) and papillary volume are of considerable clinical interest [8]. In the early 1970s, Levine introduced periodontal flap surgery with gingival fiber retention as a biologically oriented alternative to conventional resective procedures [9]. The core concept was to preserve a band of SCT fibers inserted in root cementum apical to the junctional epithelium, rather than removing all soft tissues coronal to the alveolar crest [9]. Levine proposed that maintaining these inserted collagen fibers could provide a stable connective‐tissue barrier, limit apical migration of the epithelial attachment, and support a more coronal postsurgical gingival margin [9, 10].

Subsequent clinical reports extended the use of fiber‐retention procedures to the esthetic maxillary anterior region. Dello Russo demonstrated that fiber retention could be applied successfully in patients with early to moderate periodontitis, enabling effective pocket elimination while maintaining interdental papilla height and minimizing root exposure [8]. More recently, Carnevale proposed fiber retention osseous resective surgery (FibReORS) as a novel conservative approach for eliminating shallow infrabony defects [11, 12, 13]. In contrast to classical osseous resective surgery, which uses the most apical interproximal bony crest as the reference for ostectomy, FibReORS uses the level of the connective tissue fiber attachment as the new “bottom” of the defect. Clinical follow‐up studies have suggested that shallow probing depths achieved with FibReORS can be maintained long‐term under supportive periodontal care, with low rates of disease recurrence [11, 12, 13].

Despite these advances, there remains a clinical gap in the management of esthetic anterior cases both periodontitis‐related and prosthetically driven where combined horizontal and shallow infrabony components or disharmonious gingival margins are present. To address this gap, papilla‐preserving gingival fiber‐retention osseous surgery (PP‐FibReOS) is proposed as a proof‐of‐concept variant within the broader family of fiber‐retentive resective procedures. Conceptually, PP‐FibReOS adapts Levine's gingival fiber‐retention flap to contemporary anterior indications ranging from chronic periodontitis to prosthetically driven crown lengthening, but differs from previously described approaches by (1) prioritizing preservation of de‐epithelialized SCT attached to the palatal flap, (2) intentionally constructing a double‐layered papillary complex through superimposition of a de‐epithelialized interproximal papilla beneath a buccal split‐thickness papilla, and (3) combining these soft‐tissue maneuvers with a split‐full‐split flap design and minimal ostectomy with predominately osteoplasty‐oriented osseous reshaping tailored to high‐esthetic‐risk anterior defects. The aim of this two‐case report is therefore to describe the surgical steps and 36‐month clinical outcomes of PP‐FibReOS in an advanced periodontitis case and a prosthetically driven functional crown‐lengthening case involving the maxillary anterior region (Table 1).

**TABLE 1:** Summary of case characteristics and clinical outcomes at baseline and 36‐month follow‐up.

Case No.	Age (years)	Sex	Diagnosis	Follow‐up (months)	PPD (baseline)	PPD (36 m)	CAL (baseline)	CAL (36 m)	REC (baseline)	REC (36 m)	KTW (baseline)	KTW (36 m)	Clinician‐reported esthetic score (PES)	Patient satisfaction rating
Case 1	56	M	Stage IV, Grade C periodontitis	> 36	4–7 mm	1–3 mm	3–4 mm	1–3 mm	1–2 mm	0–1 mm	7 mm	7 mm	11	10
Case 2	49	F	Supracrestal tissue attachment violation	> 36	1–3 mm	1–3 mm	None	None	None	None	5 mm	5 mm	12	10

Abbreviations: CAL, clinical attachment level; F, female; KTW, keratinized tissue width; M, male; PES, pink esthetic score; PPD, probing pocket depth; REC, gingival recession.

## 2 Clinical Technique: PP‐FibReOS

PP‐FibReOS was developed as a biologically oriented modification of osseous resective surgery that integrates the key concepts of Levine's gingival fiber‐retention flap [9, 10] and Ramfjord's [14] emphasis on preserving interproximal tissue while reshaping bone into a maintainable physiologic architecture. In PP‐FibReOS, SCT fibers are deliberately preserved and incorporated into the palatal flap rather than being left as an interproximal tissue “island,” as in Levine's original design [9], with the intent of helping control soft‐tissue position and limit postsurgical recession in the esthetic zone. At the same time, minimal interproximal soft‐tissue scalloping and osseous recontouring follow Ramfjord's principle of establishing a cleansable, physiologic contour, while respecting the coronal level of the retained SCT fiber apparatus [15, 16, 17, 18, 19]. By treating the interproximal bone connective tissue complex as a functional unit and minimizing vertical bone removal beneath the papillae, PP‐FibReOS aims to combine effective pocket elimination with preservation of interproximal soft‐tissue support, thereby reducing the risk of papillary collapse and black triangle formation while still providing the access and long‐term maintainability expected from osseous resective surgery.

### 2.1 Case Selection and Indications

PP‐FibReOS was reserved for patients in whom the soft tissue and bony architecture offered a favorable biologic starting point. Ideal candidates exhibited a thick periodontal phenotype, horizontal or only shallow angular defects, and SCT dimensions sufficient to allow fiber preservation without compromising the interproximal soft‐tissue complex. The technique was primarily considered in high‐esthetic‐risk anterior sextants and in functional crown‐lengthening situations where maintaining interdental papillae was a critical treatment objective.

Conversely, PP‐FibReOS was avoided in thin periodontal phenotypes, in the presence of deep, narrow intrabony defects, or where severe interproximal attachment loss and markedly reduced papillary support were already present. Patients who were current smokers, had poor plaque control, or displayed unstable periodontal conditions were likewise not considered suitable candidates, given the technique's reliance on stable soft‐tissue conditions and rigorous maintenance.

### 2.2 Technique Description

#### 2.2.1 Step 1: Presurgical Preparation and Anesthesia

Nonsurgical therapy (supra‐ and subgingival debridement, plaque control reinforcement, correction of local plaque‐retentive factors) is completed and residual pockets with bleeding on probing are confirmed at re‐evaluation. Standard medical and periodontal risk assessment is performed, and surgery is scheduled when inflammation is below approximately 10%. On the day of surgery, the patient rinses with 0.12%–0.2% chlorhexidine; local anesthesia is achieved (Figures 1E, 4A and 8A).

**FIGURE 1:**
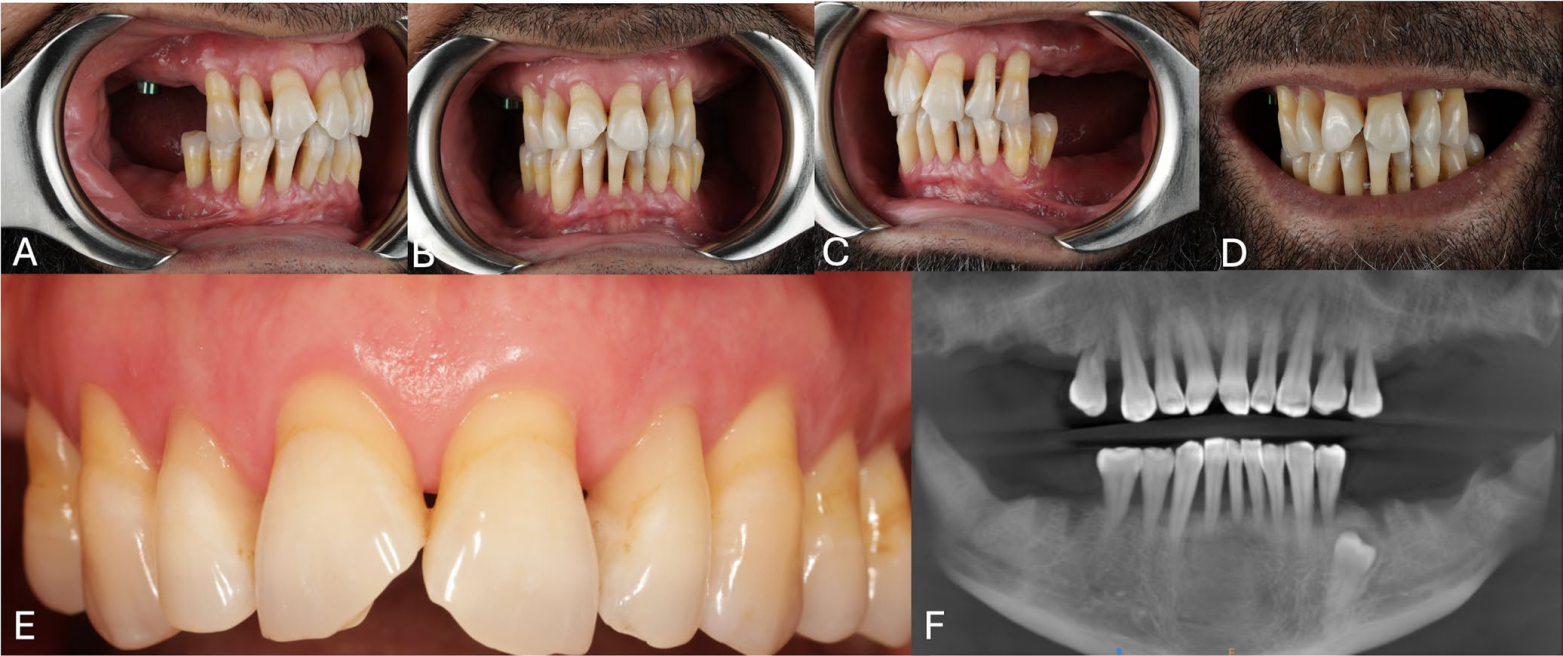
Baseline presentation of a 56‐year‐old patient diagnosed with Stage IV, Grade C periodontitis in the maxillary anterior region. (A–C) Frontal and lateral intraoral views. (D) Frontal extraoral smile views illustrating the esthetic impact of the periodontitis. (E) Close‐up view of the maxillary anterior segment demonstrating blunted interdental papillae and visible root exposure consistent with advanced attachment loss. (F) Baseline panoramic radiograph revealing generalized horizontal bone loss in the anterior sextants, compatible with Stage IV, Grade C periodontitis.

#### 2.2.2 Step 2: Buccal Split‐Full‐Split Flap Design in the Esthetic Zone

On the buccal aspect, a 0.5 mm internally beveled, scalloped incision is made across the anterior segment, positioned to preserve a band of attached gingiva and respect existing papillary form. Vertical releases are avoided when possible. A split‐thickness flap is reflected coronally, transitioning to full thickness over the crestal bone and then back to split thickness apically (split‐full‐split), allowing thinning of the marginal tissues while maintaining a SCT cuff that will be de‐epithelized and reflected with the palatal flap (Figures 2A,B and 4B).

**FIGURE 2:**
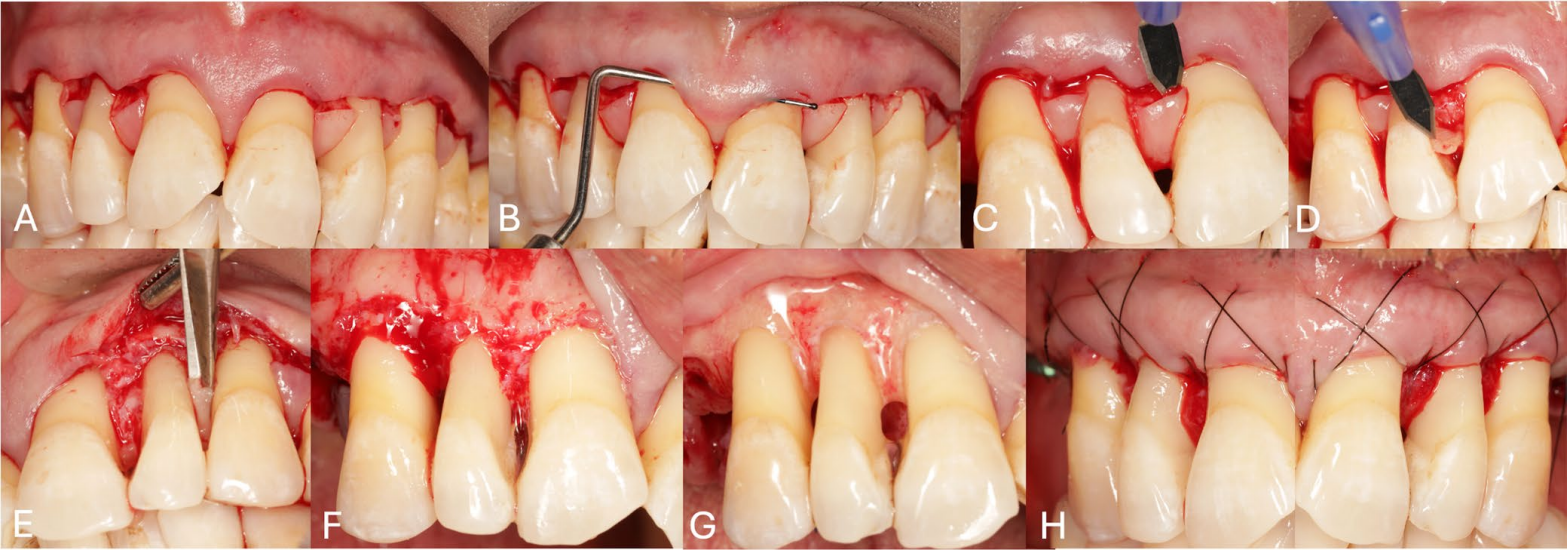
Buccal incision design for PP‐FibReOS in the maxillary anterior region. (A) A view representation of the internally beveled, scalloped buccal incision extending from canine to canine, positioned to preserve a band of attached gingiva and respect the existing papillary architecture. (B) Periodontal probe demonstrating the split‐full‐split flap design, with thinning of the marginal tissues and coronal preservation of the interdental papilla at the midline (optional step). (C–E) Steps for interproximal papilla de‐epithelialization and palatal papilla preparation in PP‐FibReOS. (C, D) With the buccal flap reflected, the interproximal papillae are selectively de‐epithelialized using an ophthalmic blade (optional) to remove only the epithelial layer while preserving the underlying connective‐tissue core. (E) Completion of papillary de‐epithelialization toward the palatal aspect using microscissors, ensuring a continuous vascular connective‐tissue bed. (F, G) Views before and immediately after osseous recontouring, illustrating a more physiologic bony architecture. (H) Final apically positioned flap over the prepared interproximal bed demonstrating the double‐layered soft‐tissue complex.

#### 2.2.3 Step 3: Palatal Full‐Thickness Flap and Papilla De‐Epithelialization

On the palatal aspect, a 0.5–1 mm internally beveled, scalloped incision is made across the midpalatal surface of the involved teeth. Only a full‐thickness flap is reflected to provide access for debridement and osseous reshaping from the palatal side. The interproximal papillae are carefully de‐epithelialized either immediately before or after flap elevation, creating a vascular connective tissue bed capable of integrating with the buccal papilla. The de‐epithelialized interproximal papilla is then gently detached from the adjacent root surfaces using a small universal curette and reflected together with the palatal flap (Figures 2C–E and 4C–E).

#### 2.2.4 Step 4: Osseous Surgery and Double‐Layered Papillary Complex

Granulation tissue and pocket epithelium coronal to the crestal bone are removed to establish a clean root surface. Because a portion of the supracrestal fiber apparatus remains attached to the palatal flap, only minimal interproximal ostectomy is performed, and osseous recontouring is limited to what is necessary to achieve physiologic architecture and optimal flap adaptation. The de‐epithelialized palatal papilla is then rotated beneath the buccal split‐thickness papilla and sutured to it apically, creating a double‐layered interproximal soft‐tissue complex designed to provide structural support for papillary volume and resist collapse of the embrasure. This double‐layered papillary complex represents the central biologic and mechanical principle of the PP‐FibReOS technique (Figures 2F,G, 4F,G).

#### 2.2.5 Step 5: Flap Repositioning, Suturing, and Postoperative Care

The buccal split‐thickness flap is adapted over the preserved connective tissue fibers and de‐epithelialized interproximal papillae and stabilized with either a single external vertical mattress or a continuous crossing‐mattress periosteal suture technique [20] (Figures 2H, 4H, 8B–D). The choice of suturing technique was adapted to the extent and configuration of the treated segment. Single external vertical mattress sutures were used for isolated or short‐span defects, where they provided secure buccolingual stabilization of the interproximal complex and allowed fine adjustment of the apical repositioning of individual papillae. In contrast, a continuous crossing mattress periosteal suture was selected for longer segments involving multiple adjacent defects, as this configuration facilitated uniform apical repositioning of the flap, distributed tension along the treated area, and secured the facial tissue positioning alongside interproximal papillae. The flap is positioned at or slightly apical to the original gingival margin, with intimate interproximal adaptation. The buccal split‐thickness flap is advanced to cover the de‐epithelialized papilla, with the buccal papillary tissue overlapping the palatal connective‐tissue papilla as extensively as is compatible with passive flap positioning. This creates a double‐layered interproximal complex in which the buccal layer provides the external contour and the palatal connective‐tissue layer serves as the internal scaffold, helping to provide a stable connective‐tissue barrier, limit apical migration of the epithelial attachment, and support a more coronal postsurgical gingival margin. Postoperative care includes chlorhexidine rinses, systemic analgesics (and antibiotics when indicated), suture removal after 7–10 days, and enrollment in a 3–6 monthly supportive periodontal care program (Figures 3A,B, 4I, 8E).

**FIGURE 3:**
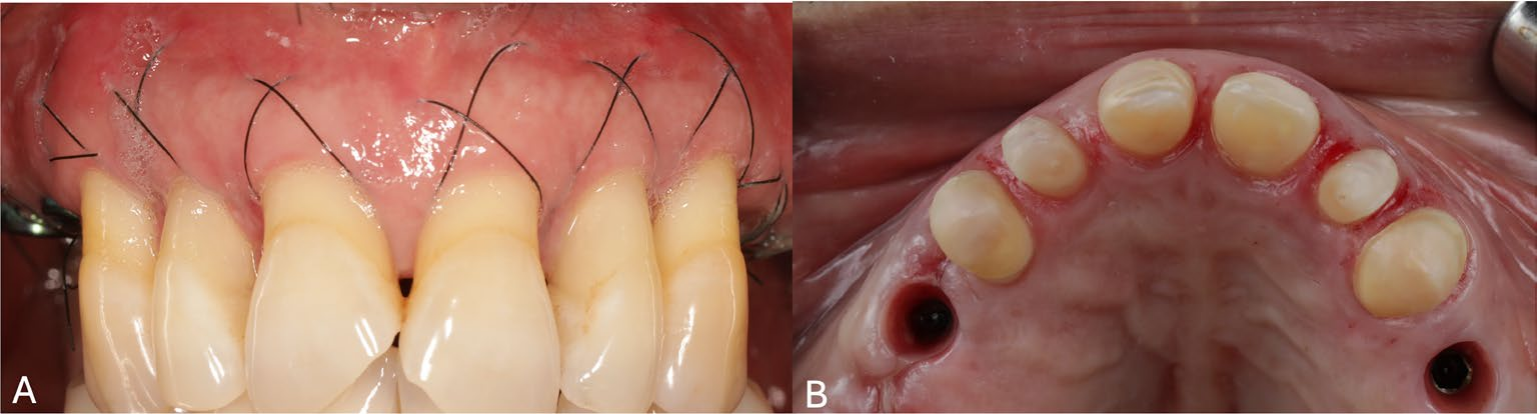
Early healing after PP‐FibReOS. (A) Ten‐day postoperative view before suture removal demonstrating uneventful early healing, with a stable flap position, re‐established marginal continuity, and initial re‐epithelialization of the embrasure areas. (B) Palatal view at 30 days showing the healed full‐thickness palatal flap with intact papillary complexes and favorable soft‐tissue adaptation around the anterior teeth and palatal defects.

**FIGURE 4:**
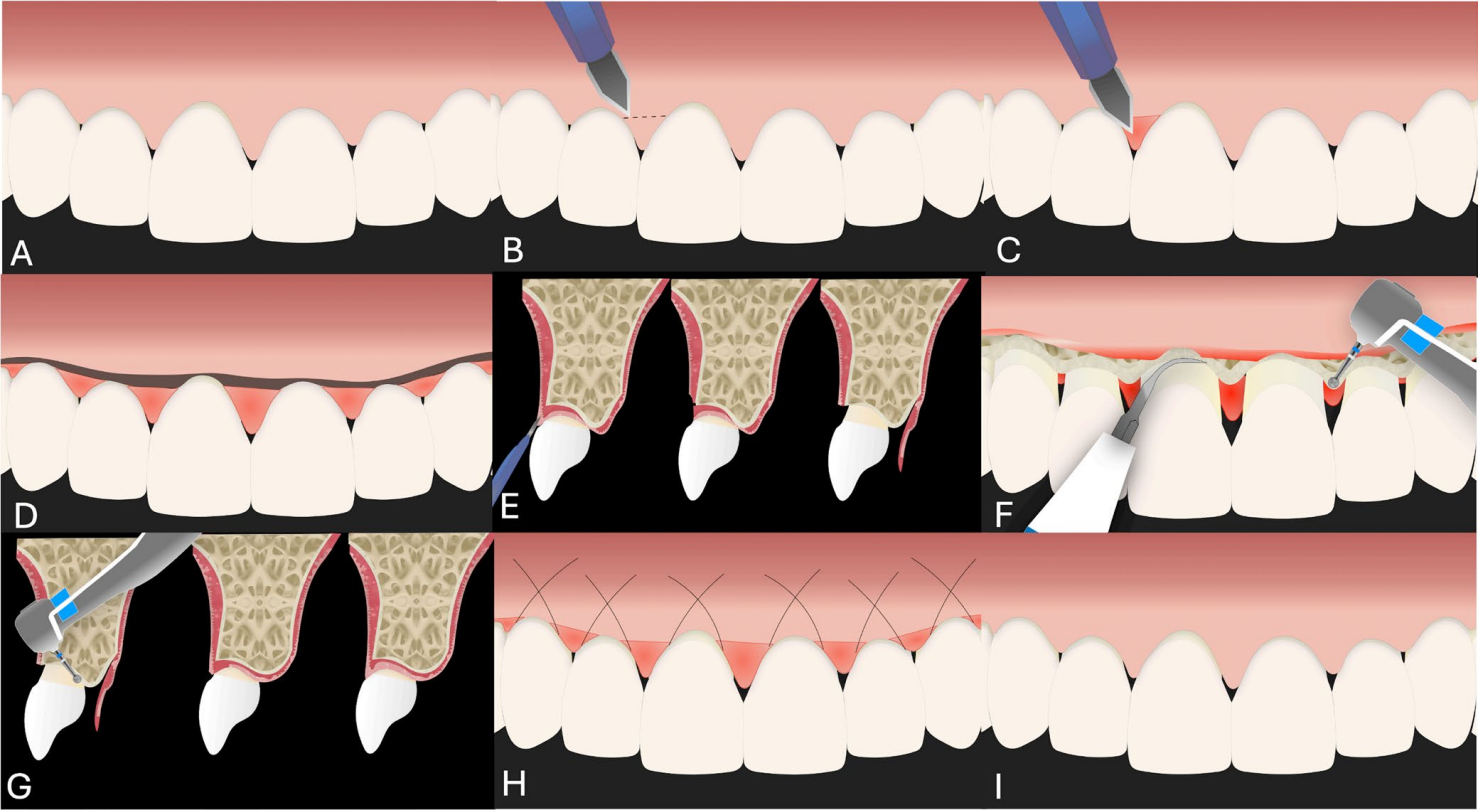
Schematic sequence of the PP‐FibReOS technique in the maxillary anterior region. (A) Baseline view of the anterior dentition and gingival architecture. (B) Design of an internally beveled, scalloped buccal incision from canine to canine, positioned to preserve a band of attached gingiva and respect the interdental papillae. (C) Completion of the buccal incision with extension into the interdental areas as needed. (D) Split‐full‐split buccal flap reflection, creating a thin, mobile marginal flap while maintaining a broader attached band apically. (E) Elevation of a full‐thickness palatal flap and selective de‐epithelialization of the interproximal papillae, preserving the supracrestal connective‐tissue fibers. (F) Osseous recontouring of the facial aspect to establish a more physiologic bony architecture and reduce angular defects. (G) Apical repositioning of the buccal flap over the rotated palatal/interproximal connective‐tissue complex, creating a double‐layered papillary bed. (H) Placement of crossing‐mattress sutures to stabilize the apically positioned flap and interproximal tissues. (I) Final schematic appearance showing re‐established scalloped gingival margins and preserved papillary volume in the esthetic zone.

**FIGURE 5:**
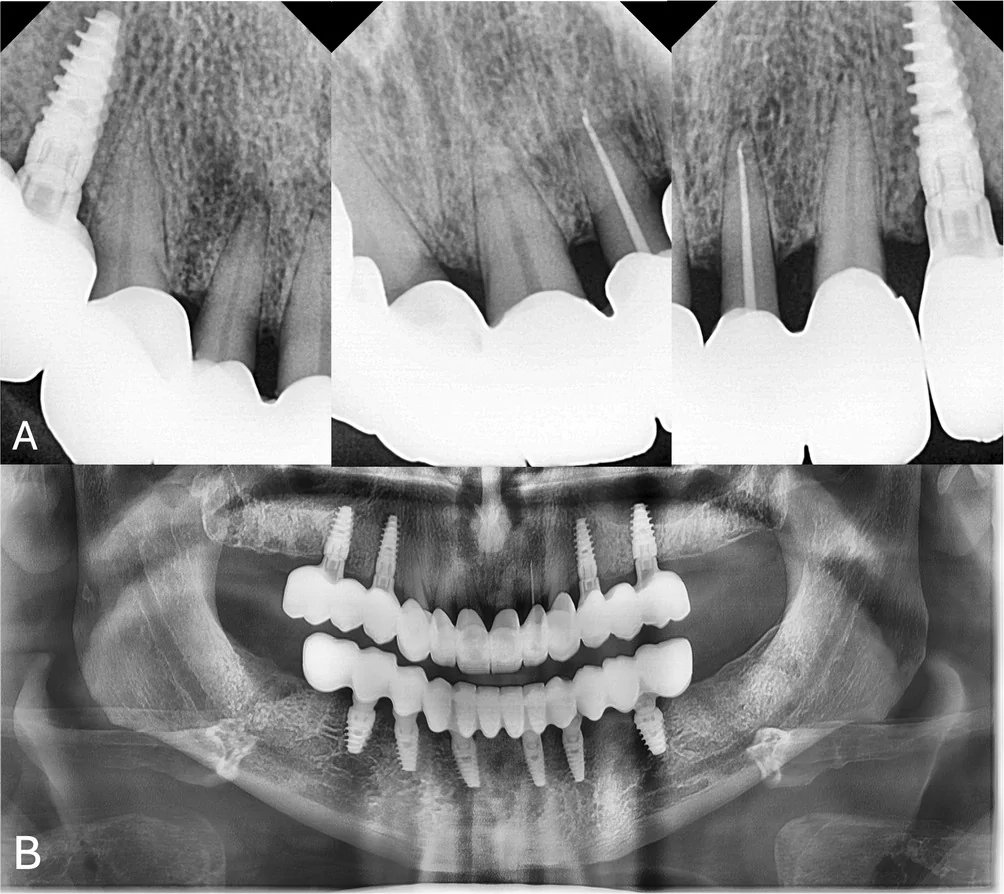
Radiographic findings at 36‐month follow‐up after PP‐FibReOS and prosthetic rehabilitation. (A) Series of periapical radiographs of the treated anterior maxillary region showing stable crestal bone levels around the natural teeth and adjacent implants, without evidence of progressive vertical bone loss or defect recurrence. (B) Panoramic radiograph at 36 months demonstrating generalized radiographic stability of the anterior sextants and restored arches, consistent with long‐term maintenance of periodontal support following the papilla‐preserving gingival fiber retention osseous surgery.

### 2.3 Case Applications

This technique was applied in two anterior maxillary situations at high esthetic risk: an advanced periodontitis case requiring pocket elimination and subsequent prosthetic rehabilitation (Figures 1, 2, 3, 4, 5, 6) and a crown‐lengthening case with supracrestal tissue attachment violation (Figures 7, 8, 9, 10). The inclusion of both a severe Stage IV Grade C periodontitis case and a prosthetically driven crown‐lengthening case reflects an intentional proof‐of‐concept design aimed at illustrating the shared indication spectrum of PP‐FibReOS rather than providing internally homogeneous outcomes. In both scenarios, the technique was applied only where a comparable set of prerequisites was present, namely, several millimeters of SCT, predominantly horizontal bone loss or a compromised interproximal papilla, and a critical need to preserve papillary volume and anterior esthetics. Nevertheless, we acknowledge that this heterogeneity limits internal consistency and does not permit firm conclusions regarding specific case types; larger series with more narrowly defined inclusion criteria will be required to delineate the most appropriate indications for PP‐FibReOS within both periodontal and prosthetically driven contexts. In addition, the restorative and prosthetic phases, including the design of provisional and definitive restorations, likely contributed to soft‐tissue maturation and stability and should be considered integral components of the overall treatment concept.

**FIGURE 6:**
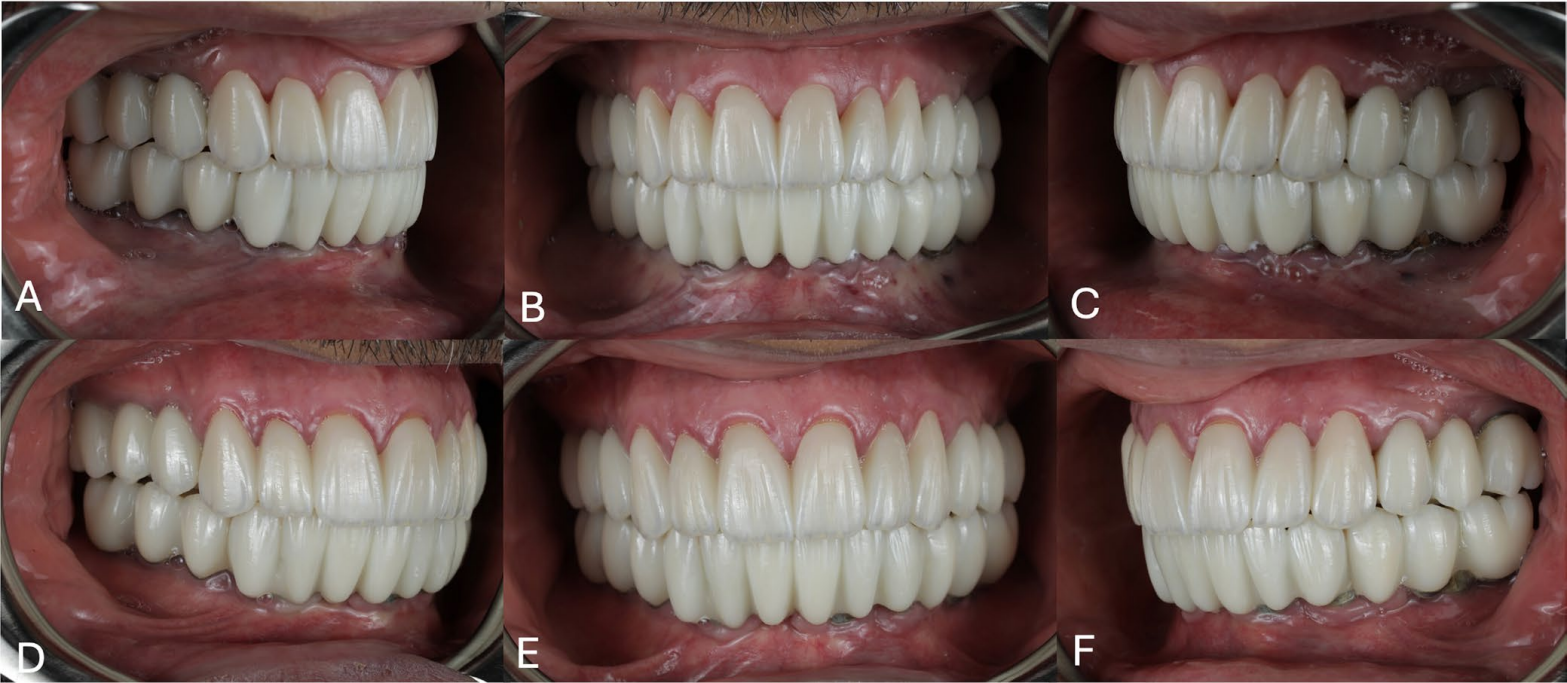
Intraoral views of the definitive prostheses at delivery and at 36‐month follow‐up after PP‐FibReOS and full‐arch rehabilitation. (A–C) Immediate postoperative views of the maxillary and mandibular definitive restorations in right lateral, frontal, and left lateral perspectives, showing harmonious tooth proportions, symmetric gingival contours, and satisfactory papillary fill in the esthetic zone. (D–F) Corresponding views at 36 months demonstrating stable soft‐tissue architecture and prosthetic integration, with preserved gingival margins except for a slight midfacial recession at the upper left central incisor, maintained papillary support with tissue rebound interproximal and absence of clinically evident black triangles, consistent with long‐term esthetic and functional stability under supportive periodontal care.

**FIGURE 7:**
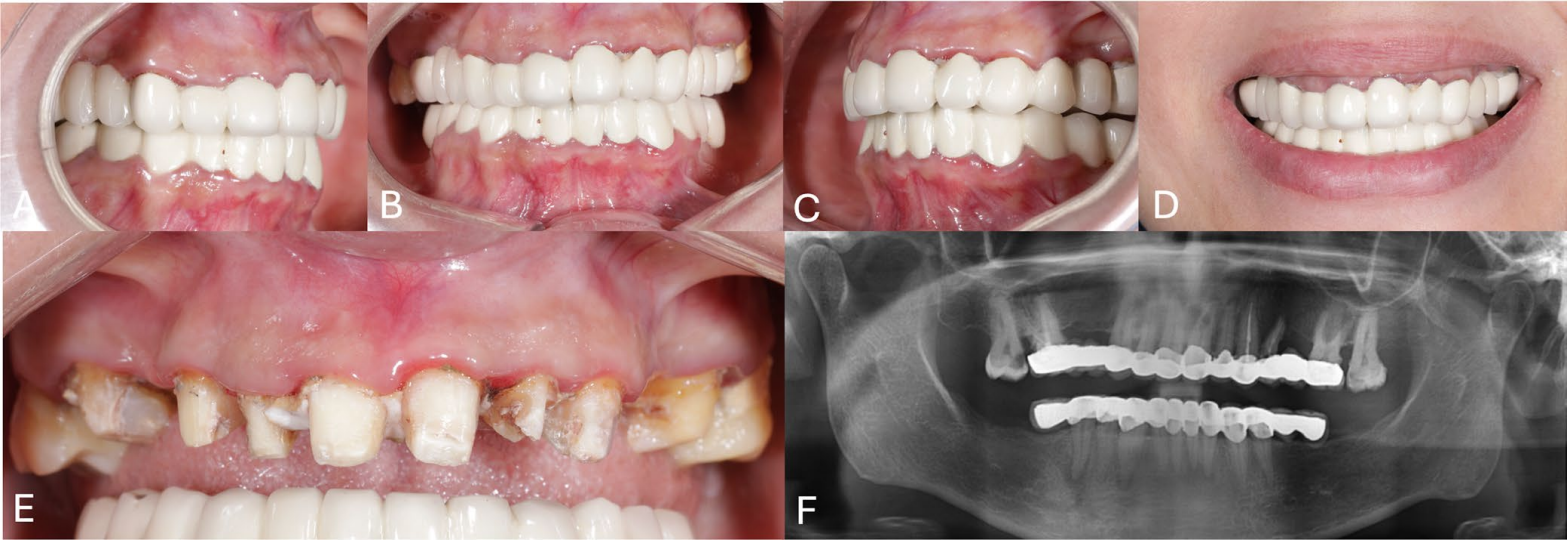
Baseline presentation of Case 2, a 49‐year‐old medically healthy female showing severe soft‐tissue violation in the maxillary anterior region, characterized by irregular gingival margins, inflamed and displaced papillae, and overcontoured fixed prostheses compromising hygiene and esthetics. (A–C) Intraoral right lateral, frontal, and left lateral views showing overcontoured fixed prostheses in the maxillary anterior region, irregular gingival margins, and inflamed, displaced papillae. (D) Extraoral smile view illustrating the unfavorable gingival display and esthetic disharmony between the prosthetic contours and the surrounding peri‐oral soft tissues. (E) Intraoral view after removal of the maxillary prosthesis, revealing carious, structurally compromised anterior abutments with advanced loss of coronal tooth structure. (F) Baseline panoramic radiograph demonstrating multiple fixed restorations with altered crown‐to‐root relationships and generalized bone loss in the anterior maxilla, consistent with advanced periodontal and prosthetic involvement.

**FIGURE 8:**
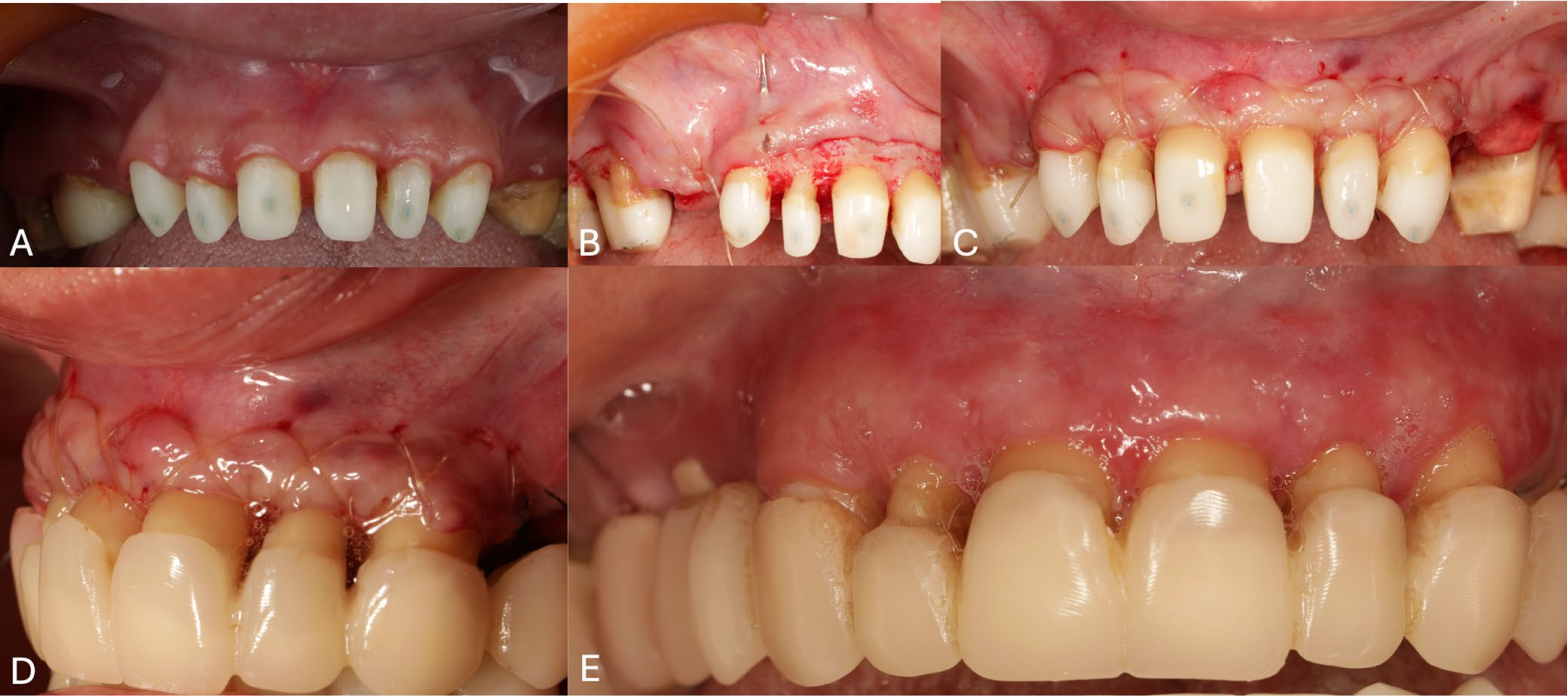
Surgical sequence and early healing for Case 2 treated with PP‐FibReOS. (A) Presurgical view after removal of the existing fixed prosthesis, showing exposed prepared abutments and irregular gingival margins in the maxillary anterior region. (B) Intraoperative view after buccal split‐full‐split flap reflection, palatal full‐thickness flap elevation, and interproximal papilla de‐epithelialization, with preservation of supracrestal connective tissue fibers and conservative osseous recontouring. (C) Immediate postoperative appearance with the buccal flap apically positioned and stabilized using a continuous crossing mattress periosteal suture technique over the rotated palatal papillae, establishing the double‐layered interproximal soft‐tissue complex. (D) Provisional restorations in place showing stable flap position and emerging papillary form. (E) Two‐week postoperative view demonstrating uneventful healing, with re‐established marginal continuity, initial papillary fill of the embrasures, and resolution of acute inflammation in the operated area.

**FIGURE 9:**
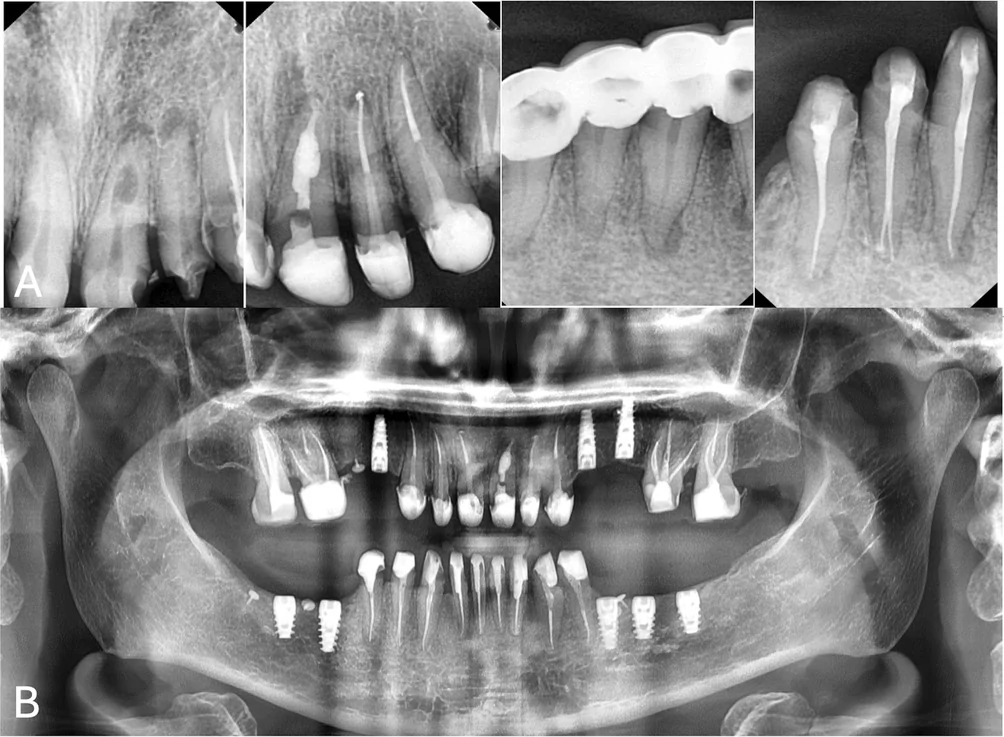
Pre‐ and posttreatment radiographic findings for Case 2. (A) Series of preoperative periapical radiographs of the maxillary anterior region showing extensive root caries, endodontically treated teeth with compromised ferrule, and advanced vertical and horizontal bone loss around the remaining abutments and anterior implants, consistent with severe periodontal and prosthetic breakdown. (B) Posttreatment panoramic radiograph following extraction of hopeless teeth, PP‐FibReOS in the anterior maxilla, strategic implant placement, and full‐arch rehabilitation, demonstrating improved distribution of abutment support and overall stabilization of the alveolar bone contours compared with the initial situation.

**FIGURE 10:**
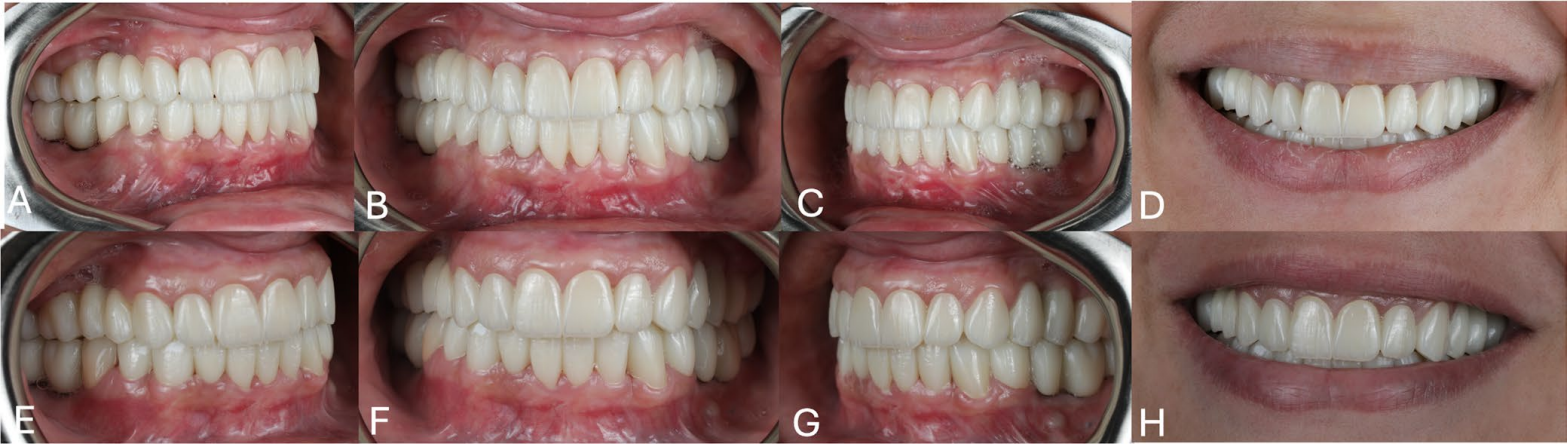
Twelve‐ and 36‐month follow‐up after PP‐FibReOS and prosthetic rehabilitation in Case 2. (A–C) Intraoral right lateral, frontal, and left lateral views at 12‐month follow‐up, showing harmonious tooth proportions, symmetric gingival contours, and satisfactory papillary fill in the anterior esthetic zone. (D) Extraoral smile view at 12 months demonstrating a natural‐appearing smile line and stable soft‐tissue support. (E–G) Corresponding right lateral, frontal, and left lateral intraoral views at 36‐month follow‐up, illustrating maintained gingival margins, preserved papillary support, and absence of black triangles or soft‐tissue collapse around the anterior teeth and implants. (H) Extraoral smile view at 36 months confirming long‐term soft‐tissue stability and high esthetic integration of the definitive prostheses.

For each treated tooth, baseline and follow‐up periodontal and esthetic parameters were recorded, including full‐mouth and site‐specific probing depths, clinical attachment levels (CAL), mid‐facial gingival recession height, width of keratinized tissue, clinician‐reported esthetic scores (PES), and patient satisfaction ratings. These measurements are summarized for both cases in Table 1.

## 3 Discussion

The present report describes papilla‐preserving gingival fiber retention osseous surgery (PP‐FibReOS) as a biologically oriented resective approach intended to eliminate anterior periodontal pockets while preserving SCT and interproximal soft‐tissue support in esthetically critical sites. In both applications of the technique, shallow residual probing depths and stable CAL were observed over 3 years without clinically relevant increases in crown length or the development of obvious black triangles. These observations suggest that, when local anatomy is favorable, PP‐FibReOS may help reconcile the traditional objectives of resective surgery (access, pocket elimination, and stable architecture) with contemporary demands for papilla preservation and maintenance of gingival symmetry around definitive restorations.

The proposed biologic rationale for PP‐FibReOS should be interpreted as hypothesis‐generating rather than as a demonstrated outcome of this report. In particular, the concept that preserved SCT functions as a biologic scaffold that supports interproximal soft‐tissue volume and limits papillary collapse, while consistent with prior fiber‐retention literature, was not directly tested in these two cases. Likewise, statements regarding the potential behavior of retained Sharpey‐type fibers and their integration into the healing connective tissue are extrapolated from histologic observations in previous studies and should not be viewed as histologic evidence specific to PP‐FibReOS [15, 16, 17, 18, 19]. Earlier fiber‐retention work by Levine et al. indicated that preserving SCT fibers can help control postsurgical marginal position and limit recession, while subsequent clinical applications in the anterior maxilla showed that, in suitably selected cases, this strategy supports pocket resolution with maintenance of papillary height. In this context, PP‐FibReOS can be viewed as a contemporary adaptation that uses these biologic principles to address combined functional and esthetic demands in the anterior maxilla, particularly where several millimeters of supracrestal tissue and favorable defect morphology are present [9, 10]. FibReORS further demonstrated that referencing the preserved fiber apparatus for osseous reshaping allows pocket elimination with more conservative vertical ostectomy [11, 12, 13]. PP‐FibReOS builds on the same preservation concept but shifts the emphasis toward a papilla‐focused, double‐layered soft‐tissue design: by retaining palatal supracrestal fibers, limiting interproximal bone removal, and overlaying a buccal split‐thickness papilla, the technique aims primarily to stabilize papillary volume and gingival symmetry while still delivering the access and shallow residual pockets expected from resective surgery.

When compared conceptually with conventional APF surgery with osseous recontouring, PP‐FibReOS may therefore offer several potential advantages in appropriately selected anterior cases. First, preservation of the interproximal supracrestal fiber apparatus and avoidance of aggressive vertical ostectomy beneath the papillae may better support long‐term papillary height, even if complete embrasure fill is not achieved in every site. Second, the split‐full‐split buccal flap design, combined with precise adaptation over the de‐epithelized SCT fiber band, appears to limit apical displacement of the gingival margin, thereby minimizing unwanted increases in clinical crown length while still achieving pocket elimination. Third, the ability to obtain shallow residual pockets while maintaining relatively stable gingival contours may mitigate the classic trade‐off between disease control and esthetic appearance that often constrains resective surgery in the anterior maxilla.

From a prosthodontic perspective, the soft‐tissue stability observed around definitive restorations in the treated cases underscores the importance of integrating fiber‐preserving resective surgery into an interdisciplinary treatment plan. Maintenance of relatively stable gingival margins and papillary form facilitated a favorable emergence profile and a natural smile line, illustrating how a carefully executed resective procedure can support restorative outcomes in patients with high esthetic demands. Even though some embrasures may not be completely filled, reducing the number and visibility of black triangles may substantially improve patient satisfaction compared with conventional resective approaches.

This report has several important limitations that warrant a cautious interpretation of the findings. First, PP‐FibReOS was applied only in carefully selected anterior cases with favorable supracrestal tissue height and defect configuration, which limits the generalizability of the results to more complex morphologies or sites with reduced attached gingiva. Second, the study lacked a parallel control group treated with conventional APF or regenerative procedures, so any apparent advantages of PP‐FibReOS remain hypothesis‐generating rather than demonstrative of superiority. Third, the small sample size and relatively short follow‐up period preclude firm conclusions about long‐term stability of papillary volume, soft‐tissue symmetry, and periodontal support. In addition, the restorative and prosthetic phases including the design of provisional and definitive restorations likely contributed to soft‐tissue maturation and stability and may be considered integral components of the overall treatment concept. Finally, the surgical protocol involved a high level of operator experience, and it is unclear whether similar outcomes could be routinely achieved in broader clinical practice without additional training and without careful case selection of patients able to maintain meticulous plaque control and comply with supportive periodontal care [21, 22]. Future studies should compare PP‐FibReOS with conventional APF and regenerative techniques in similar defect types, ideally incorporating standardized esthetic indices and patient‐reported outcomes. In that context, the adjunctive use of biologic agents may be explored to determine whether they further enhance soft‐tissue healing and maturation when combined with PP‐FibReOS [23, 24, 25].

## 4 Conclusions

Within the limitations of a two‐case proof‐of‐concept report involving anterior maxillary sites with 3‐year follow‐up, papilla‐preserving gingival fiber‐retention osseous surgery (PP‐FibReOS) may offer a feasible approach in situations where pocket reduction or functional crown lengthening must be balanced against the need to preserve papillary support in the esthetic zone. In the cases presented, conservative osseous recontouring combined with deliberate preservation and coronal repositioning of supracrestal gingival fibers was associated with stable shallow probing depths, maintenance of clinical attachment, and largely preserved interdental papillary fill, without obvious increases in clinical crown length or conspicuous black triangles at medium‐term follow‐up. These observations should be regarded as illustrative rather than definitive, and they underscore the need for larger, controlled clinical studies directly comparing PP‐FibReOS with conventional APF surgery and regenerative procedures in similar defect types, in order to clarify its indications, limitations, and long‐term predictability.

## Author Contributions

A.E.A.: conceptualization, project administration, methodology, data curation, writing – original draft, and writing – review and editing. H‐L.W.: supervision, critical review, and editing.

## Conflicts of Interest

The authors declare no conflicts of interest.

## Data Availability

The data that support the findings of this study are available from the corresponding author upon reasonable request.
